# *PTCH1*-null induced pluripotent stem cells exclusively differentiate into immature ectodermal cells with large areas of medulloblastoma-like tissue

**DOI:** 10.1007/s12672-022-00498-x

**Published:** 2022-05-27

**Authors:** Kazuaki Nagao, Chise Kato, Yu Ikemoto, Toshino Motojima, Katsunori Fujii, Akihiro Umezawa, Toshiyuki Miyashita

**Affiliations:** 1 Department of Molecular Genetics, Kitasato University Graduate School of Medical Sciences, 1-15-1 Kitasato, Minami-ku, Sagamihara, 252-0374 Japan; 2 Department of Reproductive Biology, National Center for Child Health and Development, Tokyo, 157-8535 Japan; 3 Department of Pediatrics, Motojima General Hospital, Gunnma, 373-0033 Japan; 4 Department of Pediatrics, Graduate School of Medicine, International University of Health and Welfare, Chiba, 286-8686 Japan

**Keywords:** Nevoid basal cell carcinoma syndrome, *PTCH1*, Medulloblastoma, iPSCs

## Abstract

Nevoid basal cell carcinoma syndrome (NBCCS) is an autosomal dominant disorder with an increased incidence of tumors, such as basal cell carcinomas and medulloblastomas. The *PTCH1* gene, responsible for NBCCS, suppresses the hedgehog signaling pathway, which is recognized as one of the important pathways in tumorigenesis and, thus, is a therapeutic target in cancer. In the present study, we generated *PTCH1*^−/−^ induced pluripotent stem cells (iPSCs) from NBCCS patient-derived iPSCs (*PTCH1*^+/−^) by gene editing. The proliferation of *PTCH1*^−/−^ iPSCs was accelerated due to the activation of the hedgehog signaling pathway. When *PTCH1*^−/−^ iPSCs were subcutaneously injected into immunodeficient mice, the resulting teratomas almost exclusively contained immature ectodermal lineage cells expressing medulloblastoma markers, and the percentages of the area occupied by medulloblastoma-like tissue were larger in *PTCH1*^−/−^ teratomas than in *PTCH1*^+/−^ teratomas. In contrast, in *PTCH1*^+/+^ teratomas, medulloblastoma-like tissue positive for all of these medulloblastoma markers was not observed. The present results indicate the importance of *PTCH1* in medulloblastoma formation and the suitability of these gene-edited iPSCs and *PTCH1*^−/−^ teratomas as models for the formation of tumors, such as medulloblastomas and Hh-related tumors.

**Supplementary Information:**

The online version contains supplementary material available at 10.1007/s12672-022-00498-x.

## Introduction

Nevoid basal cell carcinoma syndrome (NBCCS; OMIM 109400), also known as Gorlin syndrome, is an autosomal dominant disorder that is characterized by a variety of developmental abnormalities and an increased incidence of tumors, such as medulloblastomas, basal cell carcinomas (BCC), and keratocystic odontogenic tumors [1, 2]. The gene responsible for NBCCS is *PTCH1*, the human homolog of the *Drosophila patched* gene, located on 9q21.2 [3, 4]. This gene encodes a protein of 1,447 amino acid residues containing two large extracellular loops and 12-pass transmembrane domains that serves as a receptor for hedgehog (Hh) ligands, namely Sonic hedgehog, Desert hedgehog and Indian hedgehog. The Hh signaling pathway plays an important role in embryonic development, cell proliferation, and tumorigenesis [5, 6]. In the absence of Hh binding, PTCH1 suppresses 7-pass transmembrane protein smoothened (SMO), another component of the Hh signaling pathway. After the binding of ligands to PTCH1, the inhibitory effects of SMO are released, resulting in the activation of GLI transcription factors and alterations in target gene expression. Therefore, genetic mutations in *PTCH1* lead to the constitutive activation of signaling that results in the development of NBCCS [7–10].

The Hh signaling pathway was recently recognized as one of the important pathways for carcinogenesis and as a therapeutic target in cancer. It remains active and plays important roles in the regulation of tissue homeostasis and stem cell maintenance in adults [11]. Besides NBCCS, the up-regulation of Hh signaling has been implicated in many sporadic malignancies, including breast cancer, pancreatic cancer, lung cancer, prostate cancer, BCC, and medulloblastomas [12–15]. Vismodegib, an inhibitor of SMO, is the first oral therapeutic agent to be tested for the treatment for BCC and Sonic hedgehog subgroup medulloblastomas in adults [16, 17]. Despite positive outcomes in Phase II clinical trials, safer and less toxic drugs are required due to side effects and the acquisition of resistance after the mutation of the *SMO* gene [17, 18]. Therefore, the establishment of Hh-related tumor models is expected for the effective screening of therapeutic small chemicals.

We previously reported the formation of medulloblastoma-like tissue in teratomas generated from NBCCS patient-derived induced pluripotent stem cells (iPSCs) and the loss of heterozygosity or a second mutation in the *PTCH1* gene in the medulloblastoma, but not in the non-medulloblastoma portion of the teratoma [19]. In the present study, we disrupted a wild-type *PTCH1* allele remaining in NBCCS-iPSCs using the CRISPR/Cas9 system and injected *PTCH1*^−/−^ iPSCs into immunodeficient mice to form teratomas. The resulting tumors almost exclusively contained immature ectodermal lineage cells expressing medulloblastoma markers and may be a promising tool for the screening of small molecule drugs against Hh-related tumors.

## Materials and methods

### Ethical considerations

All experiments described below were reviewed and approved by the Institutional Review Board of Kitasato University School of Medicine, Chiba University Graduate School of Medicine and the National Center for Child Health and Development. Written informed consent was obtained from the patients. All procedures involving animals were reviewed and approved by the Institutional Animal Care and Use Committee of Kitasato University School of Medicine (approval number: 2021-099). In all experiments, the size of the tumors did not exceed 10% of body weight, which is the tumor burden permitted by the committee. All experiments were performed in accordance with the guidelines of the National Institutes of Health, the Ministry of Education, Culture, Sports, Science and Technology (MEXT) of Japan, and ARRIVE guidelines.

### Plasmid construction

In order to alter the *PTCH1* gene sequence in human iPSCs, a pair of phosphorylated and annealed oligonucleotides listed in supplementary Table S1 were cloned into the *BbsI* sites of the chimeric guide RNA and human codon-optimized Cas9 expression vector plasmids, pX330 or pX260, obtained from AddGene (https://www.addgene.org/) according to the manufacturer’s instructions.

### Antibodies

Antibodies to SSEA4 (Merck Millipore), glial fibrillary acidic protein (GFAP) (R&D Systems), α-smooth muscle actin (α-SMA) (R&D Systems), alpha fetoprotein (AFP) (R&D Systems), β-III tubulin (Promega), synaptophysin (Agilent), Ki67 (Abcam), SHH (SantaCruz), and cleaved caspase-3 (Cell Signaling Technology) were used in the present study. An Alexa488-conjugated anti-mouse goat antibody (ThermoFisher) was used as a secondary antibody for immunocytochemistry.

### Generation of iPSCs from NBCCS patients

The generation of NBCCS-specific iPSCs (NBCCS-iPSCs) from three cases, G11 (c.3130_3131dupGC), G36 (approximately 1.1-Mb deletion including the entire *PTCH1* gene), and G72 (c.274delT), was previously described [20–22]. In brief, fibroblasts were obtained from non-cancerous tissues adjacent to the cancer during surgery and infected with Sendai viruses expressing the human transcription factors OCT3/4, SOX2, KLF4, and C-MYC. The emerging colonies were picked up and expanded.

### Cell culture

Human iPSCs were maintained in StemFit AK02N human iPSC culture medium (Takara-bio) on a dish coated with iMatrix-511 matrix (Takara-bio) at 37 °C in humidified air with 5% CO_2_. Medium was changed every other day. Human iPSC colonies comprising closely packed cells were dissociated with a 1:1 solution of TrypLE reagent (ThermoFisher) and DPBS (ThermoFisher) containing 0.5 mM EDTA after a 2 h treatment with 10 μM Y-27632 (WAKO chemical), and then scraped. Dissociated cells were seeded at a density of 1.3 × 10^3^ cells/cm^2^ and cultured in medium supplemented with 10 μM Y-27632 until the next day.

### Editing of the *PTCH1* gene in NBCCS-iPSCs

In total, 2 × 10^5^ dissociated NBCCS-iPSCs were transfected with 1 μg of the CRISPR/Cas9 vector plasmids, pX330-G11mt or pX330-G11wt for G11-iPSC, pX330-G36wt1 or pX330-G36wt2 for G36-iPSC, and pX330-G72wt or pX260-G72mt for G72-iPSC, and 1 μg of the puromycin expression vector, pENTR-lox-Puro [23], using the 4D Nucleofector device (program CB-150) and P4 Primary Cell 4D-Nucleofector solution (both from Lonza) in the 20-μl format, and then plated on a 100-mm dish coated with iMatrix-511 matrix. Forty-eight hours after transfection, iPSCs were treated with 0.75 μg/ml puromycin for 16 h and cultured for 2 weeks. Each colony was split in two, with one half being maintained in culture. The other half was used for genotyping. Cells were lysed in lysis buffer (65 mM Tris–HCl pH8.0, 15.3 mM ammonium sulfate, 1 mM 2-melcaptoethanol, 1 mM EDTA, 0.5% Triton-X100, and 3 μg/ml proteinase K) at 55 °C for 2 h, and then incubated at 95 °C for 12 min to inactivate proteinase K. Extracted genomic DNA was used for PCR direct sequencing as a template. The primers used for PCR direct sequencing are listed in supplementary Table S2.

### Immunocytochemistry of iPSCs

In total, 2 × 10^3^ dissociated iPSCs were plated on the Nunc Lab-Tek Permanox 4-well slide chamber (Merck Millipore) coated with iMatrix-511. Ninety-six hours later, cells were fixed with PBS containing 4% paraformaldehyde at 4 °C for 1 h and permeabilized with PBS containing 0.3% Triton-X100 at room temperature for 1 h. After a treatment with the blocking solution, Block ACE (Bio-Rad), at room temperature for 1 h, cells were stained with the primary antibody followed by the Alexa488-conjugated secondary antibody. Cells were observed under the confocal microscope, LSM710 (Zeiss).

### Cell proliferation assay

A cell proliferation assay was performed using the BrdU cell proliferation ELISA kit (Abcam) according to the manufacturer’s instructions. In brief, dissociated 400 iPSCs were plated on a 96-well plate coated with iMatrix-511. After 24, 48, 72, 96, 120 or 144 h of culture, cell were labeled with BrdU for 24 h, and fixed. Then, cells were treated with anti-BrdU antibody, peroxidase-conjugated secondary antibody, and TMB peroxidase substrate. Rates of BrdU incorporation were measured by reading the plate using the SpectraMax M2 spectrophotometer (Molecular Devices) at a wavelength of 450 nm.

### Teratoma formation

Confluent human iPSCs cultured in a 60-mm dish were dissociated, suspended in 400 μl of a 1:1 solution of DPBS and Matrigel hESC-Qualified Matrix (Corning), and then subcutaneously injected into the dorsal flanks of CB17/IcrJcl-*Prkdc*^*scid*^ immunodeficient mice (CLEA Japan, Inc.). Eight to twelve weeks after the injection, tumors were dissected and fixed in Maskedform (Japan Tanner). Paraffin-embedded tissue was sliced and stained with hematoxylin and eosin.

### Histological analysis of teratoma

Hematoxylin and eosin staining was conventionally performed. Immunohistochemistry was conducted using a combination of the microwave oven heating and Histofine Simple Stain MAX-PO (Nichirei Bioscience) methods. In brief, slices were treated by microwave oven heating in 10 mM citrate buffer (pH6.0) or 10 mM Tris–EDTA buffer (pH9.0) for 15 min for antigen retrieval after deparaffinization, and incubated at 4 °C overnight with optimized dilutions of primary antibodies. Slice images were obtained using the microscope, BZ-9000 (Keyence), and combined using the software, BZ-H4XD (Keyence), to create composite images. In order to analyze the rate of apoptosis, the images of neuroepithelium including neural tubes were obtained using × 20 objective lens and counted cleaved caspase-3-positive cells in each image.

### Quantitative RT-PCR

Quantitative RT-PCR was performed as described previously [23]. In brief, 5 µg of total RNA was reverse-transcribed and the resulting cDNA was used as a template for quantitative PCR. Primers used in the analysis are listed in supplementary Table S2. *GAPDH* served as the endogenous control. The expression levels of the Hh target genes were quantified using the Pfaffl analysis method [24].

### Statistical analysis

Data are represented as means ± standard errors of the mean (SEM), and were compared using the Student’s *t*-test (Fig. 2) or Welch’s t-test (Fig. 4c).

## Results

### Generation of *PTCH1*^−/−^ iPSCs from NBCCS-iPSCs

**Fig. 1:**
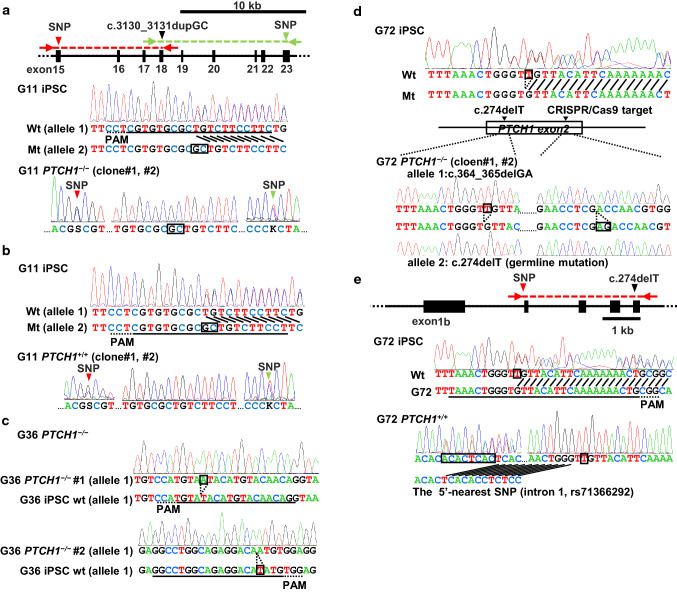
Schematic presentation of *PTCH1*-edited NBCCS iPSCs. **a**, **b** The disruption of the wild-type allele **a** and the correction of the mutated allele to the wild type **b** in G11-iPSC. The red and green arrowheads in the top panel indicate the locations of the nearest up- and downstream SNPs, respectively. The red and green arrows indicate the positions of the primer pairs used for PCR direct sequencing. **c** The disruption of the wild-type *PTCH1* allele in G36-iPSC. Note that the entire *PTCH1* on allele 2 was deleted in G36. **d**, **e** The disruption of the wild-type allele **d** and the correction of the mutated allele **e** in G72-iPSC. CRISPR/Cas9 target sequences and the protospacer adjacent motifs (PAM) were indicated by underlines and dotted underlines, respectively

In order to establish the Hh signaling-related tumor model, we disrupted the wild-type *PTCH1* allele remaining in three NBCCS-derived iPSC lines, G11-iPSC, G36-iPSC, and G72-iPSC [21, 22], by introducing the CRISPR/Cas9 expression vector, which targets the wild-type *PTCH1* sequence. We obtained two clones from each parental iPSC, named G72 *PTCH1*^−/−^, G36 *PTCH1*^−/−^, and G11 *PTCH1*^−/−^ (Fig. 1, Table 1).

**Table 1:** *PTCH1* mutations of gene-edited and parental iPSCs

Name of iPSCs	Allele 1	Allele 2
G11	wt	c.3130_3131dupGC
G11 *PTCH1*^−/−^#1	c.3130_3131dupGC	c.3130_3131dupGC
G11 *PTCH1*^−/−^#2	c.3130_3131dupGC	c.3130_3131dupGC
G11 *PTCH1*^+/+^#1	wt	wt
G11 *PTCH1*^+/+^#2	wt	wt
G36	wt	large deletion
G36 *PTCH1*^−/−^#1	c.570_571insA	large deletion
G36 *PTCH1*^−/−^#2	c.1207delT	large deletion
G72	wt	c.274delT
G72 *PTCH1*^−/−^#1	c.364_365delGA	c.274delT
G72 *PTCH1*^−/−^#2	c.364_365delGA	c.274delT
G72 *PTCH1*^+/+^	wt	wt

In G11-*PTCH1*^−/−^ iPSCs, the wild-type *PTCH1* sequence was eliminated, whereas the heterozygosity of the nearest SNPs was maintained (Fig. 1a, lower panel). Deletions between these SNP sites were not detected by PCR direct sequencing (data not shown). These results indicated that the wild-type *PTCH1* allele (allele 1) was edited into an identical sequence with a germline mutation via homologous recombination using allele 2.

To confirm the disruption of the wild-type *PTCH1* allele in G72-iPSC, we performed TA cloning to select clones in which only the wild-type *PTCH1* allele was mutated because the CRISPR/Cas9 target sequence may edit wild-type and mutated alleles. Two G72-*PTCH1*^−/−^ clones coincidently obtained the same mutation, c.364_365delGA, on allele 1 (Fig. 1d, lower panel).

In G36-iPSC, the wild-type allele (allele 1) was selectively detected by PCR due to the entire deletion of the *PTCH1* allele in allele 2. The remaining allele was efficiently disrupted by c.570_571insA or c.1207delT in clones 1 and 2, respectively (Fig. 1c).

To produce *PTCH1*^+/+^ clones from G11-iPSC, mutated *PTCH1* allele-specific CRISPR/Cas9 vectors were transfected into G11-iPSC. Two clones were obtained in which the mutated allele (allele 1) was converted into the wild-type sequence. The possibility of the deletion of allele 2 was excluded by PCR direct sequencing and the heterozygosity of nearby SNPs (Fig. 1b, lower panel).

To correct the mutated *PTCH1* allele in G11-iPSC, we employed the pX260 vector, which recognizes a target sequence of 30 bp in length [25] instead of pX330 because we were unable to identify a protospacer adjacent motif within 20 bp around the germline mutation. The possibility of the deletion of allele 2 was excluded by PCR direct sequencing and the heterozygosity of the nearby PNP of the in/del type, rs71366292 (Fig. 1e, lower panel). The genotypes of the edited clones are listed in Table 1. The expression of pluripotent markers, such as *SSEA4*, *OCT3/4*, *NANOG*, and *SOX2*, were confirmed by immunofluorescence and RT-PCR after gene editing (Fig. S1).

### Characterization of ***PTCH1***^−/−^ iPSCs

**Fig. 2:**
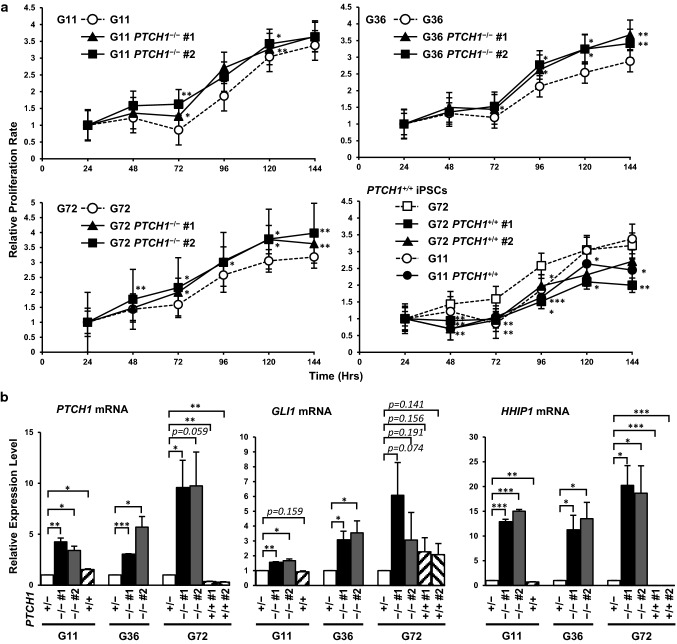
Hh signaling was accelerated in *PTCH1*^−/−^ iPSC lines. **a** Cell proliferation assay of *PTCH1*-edited iPSCs. Eight hundred cells were plated on a 96-well plate and cell numbers were measured every 24 h using the BrdU incorporation assay. **b** The expression levels of the Hh-signaling target genes, *PTCH1*, *GLI1*, and *HHIP1* were normalized by *GAPDH* mRNA levels. Data are presented as means ± SEM (*n* = *3*). *: *p* < 0.05, **: *p* < 0.01, ***: *p* < 0.001

We investigated whether Hh signaling was accelerated in *PTCH1*-edited iPSCs by measuring BrdU incorporation into newly synthesized DNA and by RT-qPCR of Hh target genes. All *PTCH1*^−/−^ iPSC clones proliferated more rapidly than parental NBCCS-iPSCs. In contrast, *PTCH1*^+/+^ clones proliferated more slowly than parental cells. These results are consistent with activated and inhibited Hh signaling in *PTCH1*^−/−^ iPSCs and *PTCH1*^+/+^ iPSCs, respectively (Fig. 2a). The expression levels of Hh-signaling target genes, such as *PTCH1*, *GLI1*, and *HHIP1*, were also investigated in these iPSCs (Fig. 2b). The expression of *PTCH1* and *HHIP1* was significantly stronger and weaker, respectively, in *PTCH1*^−/−^ clones and *PTCH1*^+/+^ clones than in parental iPSCs. Changes in the expression of *GLI1* were less evident than those of other target genes.

### Teratoma formation

**Fig. 3:**
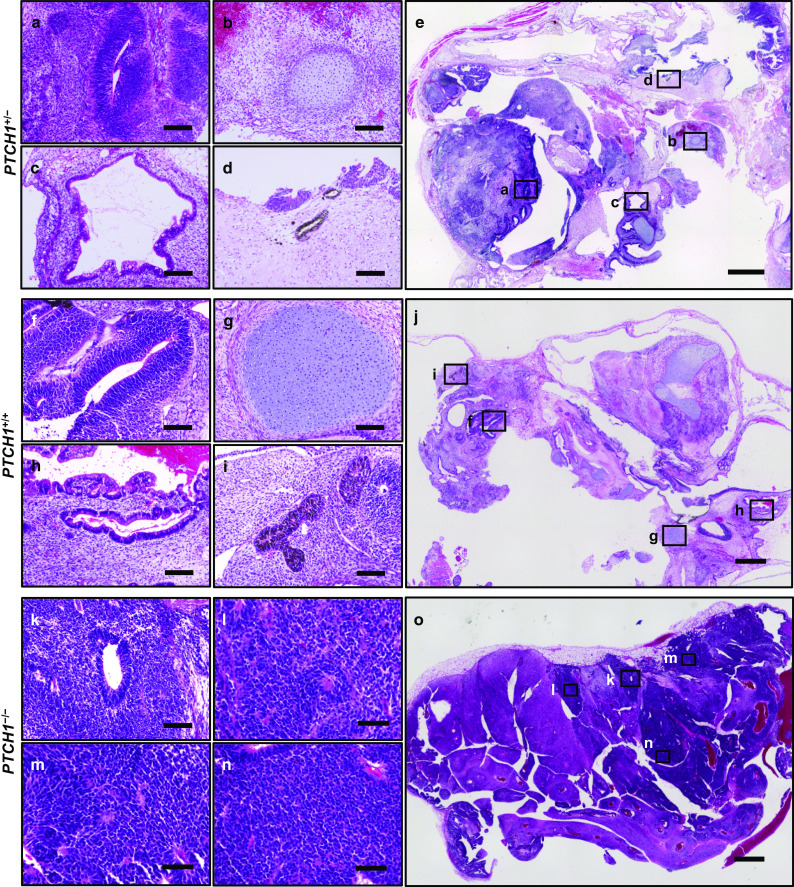
HE staining of *PTCH1*^+/−^, *PTCH1*^+/+^, and *PTCH1*^−/−^ teratomas. HE staining of a *PTCH1*^+/−^ teratoma (G11-NBCCS) (a-e), *PTCH1*^+/+^ teratoma (G11 *PTCH1*^+/+^) (**f**–**j**), and *PTCH1*^−/−^ teratoma (G36 *PTCH1*^−/−^) (**k**–**o**). Neural tubes (**a**, **f**, **k**), cartilage (**b**, **g**), the intestine (**c**, **h**), and melanocytes **d**, **i** are shown. Whole-slice images of teratomas are shown in e, **j**, and **o**. Higher magnifications of the areas indicated by rectangles in **e**, **j**, and **o** are shown in a-d, f-i, and k-n. Scale bars: 100 μm in **a**–**d**, **f**–**i**, and **k**, 1 mm in **e**, **j**, and **o**, 60 μm in **l**–**n**

We subcutaneously transplanted these iPSCs into the dorsal flanks of immunodeficient mice to generate teratomas. As we reported previously [19], paraffin-embedded sections of the resulting tumors contained the components of the three germ layers, such as the neural tube, cartilage, and the intestine, in teratomas generated from parental NBCCS-iPSCs as well as gene-edited *PTCH1*^+/+^ iPSCs (Fig. 3a–c, f–h). In addition, we confirmed the presence of melanocytes as a marker for highly differentiated ectodermal cells [26] in these teratomas (Fig. 3d, i). On the other hand, *PTCH1*^−/−^ teratomas frequently lacked mesodermal and/or endodermal components. Among the 10 *PTCH1*^−/−^ teratomas investigated, only one had all 3 germ layers, whereas three had ectodermal and endodermal tissues, and the remaining 6 teratomas only contained the ectodermal component (Fig. 3k, Table 2). In addition, no melanocytes were found in *PTCH1*^−/−^ teratomas.

**Table 2:** Numbers of teratomas with 3 germ layer components and melanocytes

	*PTCH1*^+/+^	*PTCH1*^+/−^	*PTCH1*^−/−^
Neural tube	5/5	6/6	10/10
Cartilage	5/5	5/6	1/10
The intestine	5/5	6/6	3/10
Melanocytes	4/5	4/6	0/10

**Fig. 4:**
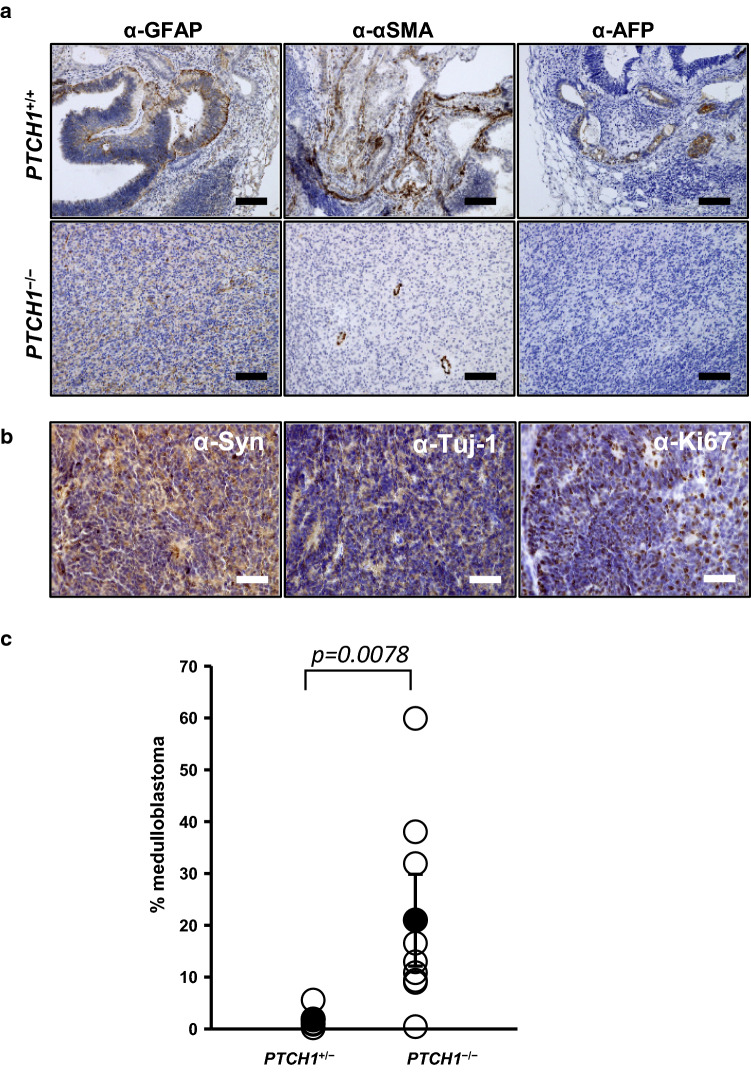
Immunohistological analysis of marker proteins in teratomas. **a** The expression of the germ layer marker proteins, GFAP, αSMA, and AFP, was immunohistochemically analyzed in a *PTCH1*^+/+^ teratoma (G11 *PTCH1*^+/+^) (upper column) and *PTCH1*^−/−^ teratoma (G72 *PTCH1*^−/−^) (lower column). Scale bar: 100 μm. **b** Expression of the marker proteins for medulloblastoma. A series of sections of a *PTCH1*^−/−^ teratoma (G36 *PTCH1*^−/−^) were stained with anti-synaptophysin, anti-βIII tubulin, and anti-Ki67 antibodies. Scale bar: 60 μm. **c** Medulloblastomas occupied a larger area in *PTCH1*^−/−^ teratomas than in *PTCH1*^+/−^ teratomas. The percentage of areas of medulloblastoma-like tissues positive for βIII tubulin, synaptophysin, and Ki67 was calculated in *PTCH1*^+/−^ and *PTCH1*^−/−^ teratomas. Closed circles and error bars represent the mean ± SEM (*PTCH1*^+/−^; *n* = *5, PTCH1*^−/−^; *n* = *9*). Open circles represent each value

We then confirmed these results by immunohistochemistry. We stained the marker proteins of the three germ layers: GFAP for the ectoderm, α-SMA for the mesoderm, and AFP for the endoderm (Fig. 4a). The *PTCH1*^+/+^ and *PTCH1*^+*/−*^ teratomas were positive for all of these marker proteins. In contrast, in *PTCH1*^−/−^ teratomas, GFAP was positive in most areas, whereas α-SMA-positive signals were only observed in perivascular cells, and AFP was negative. These results indicate that *PTCH1*^−/−^ iPSCs predominantly differentiate into ectodermal cells and remain in an undifferentiated state in vivo.

In addition, medulloblastoma-like tissues were frequently observed in *PTCH1*^−/−^ teratomas (Fig. 3l–n). These medulloblastoma-like tissues were confirmed to be strongly positive for medulloblastoma markers, such as βIII tubulin (Tuj1), synaptophysin, and Ki67 (Fig. 4b). The percentages of the area occupied by medulloblastomas were significantly larger in *PTCH1*^−/−^ teratomas than in *PTCH1*^+/−^ teratomas (Fig. 4c, Fig. S2). These results support the suitability of *PTCH1*^−/−^ teratomas as a model for Hh-related tumors, including medulloblastomas. In contrast, no medulloblastoma-like tissue positive for medulloblastoma markers was observed in teratomas generated from gene-edited *PTCH1*^+/+^ iPSCs (Fig. 3f–j), demonstrating that the rescuing a mutated allele in *PTCH1*^+*/−*^ iPSCs inhibited medulloblastoma formation.

**Fig. 5:**
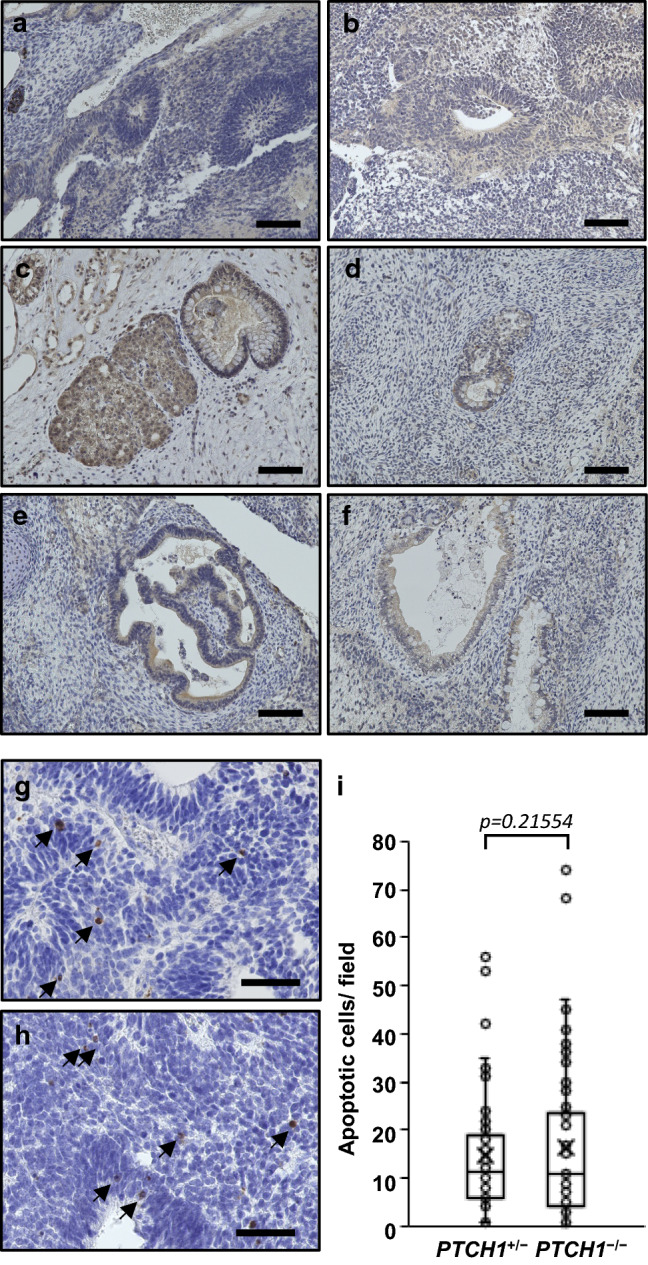
The SHH expressions and apoptotic cells in teratomas. **a**–**f** Immunohistological staining of SHH. SHH was expressed in neural tissues (**a**, **b**), glands **c**, **d** and intestinal epithelium **e**, **f** in *PTCH1*^+/−^ **a**, **c** and **e** and *PTCH1*^−/−^ teratomas (**b**, **d** and **f**). **g**–**i** Apoptosis in *PTCH1*^+/−^ **g** and *PTCH1*^−/−^ teratoma (**h**). Cleaved caspase-3 positive apoptotic cells were indicated by allows. Numbers of apoptotic cells counted in each field were displayed in the boxplot (**i**). Open circles and cross signs indicate each value of apoptotic cells and the averages, respectively (*PTCH1*^+/−^; *n* = 55, *PTCH1*^−/−^; *n* = 85). Scale bars: 100 μm in **a**–**f**, 50 μm in **g** and **h**

PTCH1 has not only been described as an Hh receptor and inhibitor of SMO, but also other functions have been unveiled (e.g. the pro-apoptotic signaling coming from the PTCH1 receptor in the absence of the Hh ligand) [27]. We investigated the expression levels of *SHH* in the iPSC lines by RT-PCR, and explored the expression of SHH protein and the rate of apoptosis in teratomas using anti-SHH and anti-cleaved caspase-3 antibodies, respectively. Only two G72-*PTCH1*^−/−^-derived iPSC lines expressed *SHH*, in which the expression levels were very low (*SHH Ct* > 28 vs. *GAPDH Ct* < 18) (Fig. S3). Regardless of *SHH* expressions in limited lines of original iPSCs, the SHH protein was weakly, but widely expressed at various tissues such as intestinal epitheliums, gland-like tissues, and neural tissues in *PTCH1*^+/−^ teratomas and in *PTCH1*^−/−^ teratomas (Fig. 5a–f). Apoptotic cells were found in *PTCH1*^+/−^ and in *PTCH1*^−/−^ teratomas, but there was no statistical difference of the apoptotic rate between two genotypes (Fig. 5g–i), suggesting the apoptosis in these teratoma was induced by ligand-free PTCH1-independent fashion.

## Discussion

Disease-specific iPSCs from patients with germline mutations are useful for understanding the initiation and progression of genetic disorders and examining relevant therapeutic targets and reagents. We previously demonstrated that when NBCCS-iPSCs were injected into immunodeficient mice, they developed teratomas containing small fractions of medulloblastoma-like tissues. These tissues carried secondary mutations in *PTCH1*, such as LOH and small insertions/deletions [19]. Previous studies reported the formation of the Hh subgroup of medulloblastomas by injecting neuroepithelial stem cells derived from NBCCS-iPSCs into the cerebellum [28, 29]. These findings prompted us to generate iPSCs in which both of the *PTCH1* alleles were mutated and test their ability to form tumors.

Although teratomas from parental NBCCS-iPSCs (*PTCH1*^+/−^ iPSCs), as well as gene-edited *PTCH1*^+/+^ iPSCs, contained all components of the three germ layers, those from gene-edited *PTCH1*^−/−^ iPSCs predominantly differentiated into ectodermal tissues. Moreover, highly differentiated ectodermal cells, such as melanocytes, were rarely observed in *PTCH1*^−/−^ iPSC-derived teratomas.

As described above, we previously reported that teratomas derived from NBCCS-iPSCs *(PTCH1*^+/−^) generated medulloblastoma-like tissues [19]. The major difference between teratomas from NBCCS-iPSCs and gene-edited *PTCH1*^−/−^ teratomas is that the latter mostly comprise ectodermal tissues and markedly larger areas were occupied by medulloblastoma-like tissues in the latter than in the former.

Hh signaling is reported to be involved in the differentiation of pluripotent stem cells into the neuroectodermal lineage in addition to the differentiation and proliferation of neural stem cells in mice and humans. Mouse and human pluripotent stem cells were previously induced to motor neuron progenitors or midbrain-specific neurons in the presence of Hh proteins [30, 31]. On the other hand, *Smo*^−/−^ or *Ihh*^−/−^ mouse ES cells failed to generate embryoid bodies containing neuroectodermal cells and nestin-positive neural stem cells [32]. Our results showing that *PTCH1*^−/−^ teratomas predominantly consisted of neuroectodermal cells are consistent with these findings. Importantly, the absence of medulloblastoma in teratomas generated from gene-edited *PTCH1*^+/+^ iPSCs confirmed the idea that *PTCH1* is indeed a driver gene in the formation of medulloblastomas observed in our study.

Apoptosis has a profound effect on tumor formation. Whereas PTCH1 was reported to induce apoptosis in the absence of its ligands, such as SHH, there was no statistical difference of the apoptotic rate between *PTCH1*^+/−^ and *PTCH1*^−/−^ teratomas in this study. The extensive expression of SHH protein in *PTCH1*^+/−^ teratoma and presence of apoptotic cells in *PTCH1*^−/−^ teratoma also support the idea that apoptosis both in *PTCH1*^+/−^ and *PTCH1*^−/−^ teratomas are induced by a PTCH1-independent manner. Since several groups have been reported that proapoptotic protein, BAX, expressed frequently in immature teratomas [33, 34], BAX expression possibly accounts for the induction of apoptosis in *PTCH1*^+/−^ and *PTCH1*^−/−^ teratomas.

BCC, the most frequent neoplasia in NBCCS, is also accompanied by secondary mutations in the wild-type *PTCH1* gene [12, 35]. However, the pathogenic characteristics of BCC, e.g. peripheral palisading and stromal retraction, were not observed in *PTCH1*^−/−^ teratomas in the present study. Since the onset of BCC is markedly later than that of medulloblastoma (the mean onset ages of BCC and medulloblastoma are 37.4 and 1.8 years, respectively, in the Japanese population [36]), the in vitro differentiation of iPSCs into keratinocytes, the primary component of the epidermis, before a subcutaneous injection may be required to generate BCC.

Since *PTCH1*^−/−^ iPSCs are one step closer to tumor formation according to the Knudson’s two-hit hypothesis [37], these gene-edited iPSCs may be a good model for the formation of medulloblastoma in NBCCS and may also be useful for drug screening to identify personalized treatments for this tumor type.

## Supplementary Information

Supplementary file1 Table S1. Oligonucleotides used for CRISPR/Cas9 vector construction. (XLS 33 KB)

Supplementary file2 Table S2. Oligonucleotides used for PCR. (XLS 36 KB)

Supplementary file3 Fig. S1. *PTCH1*-edited NBCCS iPSCs expressed pluripotency markers. (PDF 1341 KB)

Supplementary file4 Fig. S2. Whole-slice images and portions of medulloblastomas in *PTCH1*^+/−^ and *PTCH1*^−/−^ teratomas. (PDF 1070 KB)

Supplementary file5 Fig. S3. The expression of SHH in iPSCs. (PDF 783 KB)

## Data Availability

The data that support the findings of this study are available from the corresponding author, KN, upon reasonable request.
