# Supplementary material for: PTCH1-null induced pluripotent stem cells exclusively differentiate into immature ectodermal cells with large areas of medulloblastoma-like tissue
Source: Discov Oncol. 2022 May 27;13:36. doi: 10.1007/s12672-022-00498-x (PMC9135936; doi:10.1007/s12672-022-00498-x)
Supplement: Supplementary file 3 — Supplementary file3 Fig. S1. PTCH1-edited NBCCS iPSCs expressed pluripotency markers. (PDF 1341 KB) [file 12672_2022_498_MOESM3_ESM.pdf]

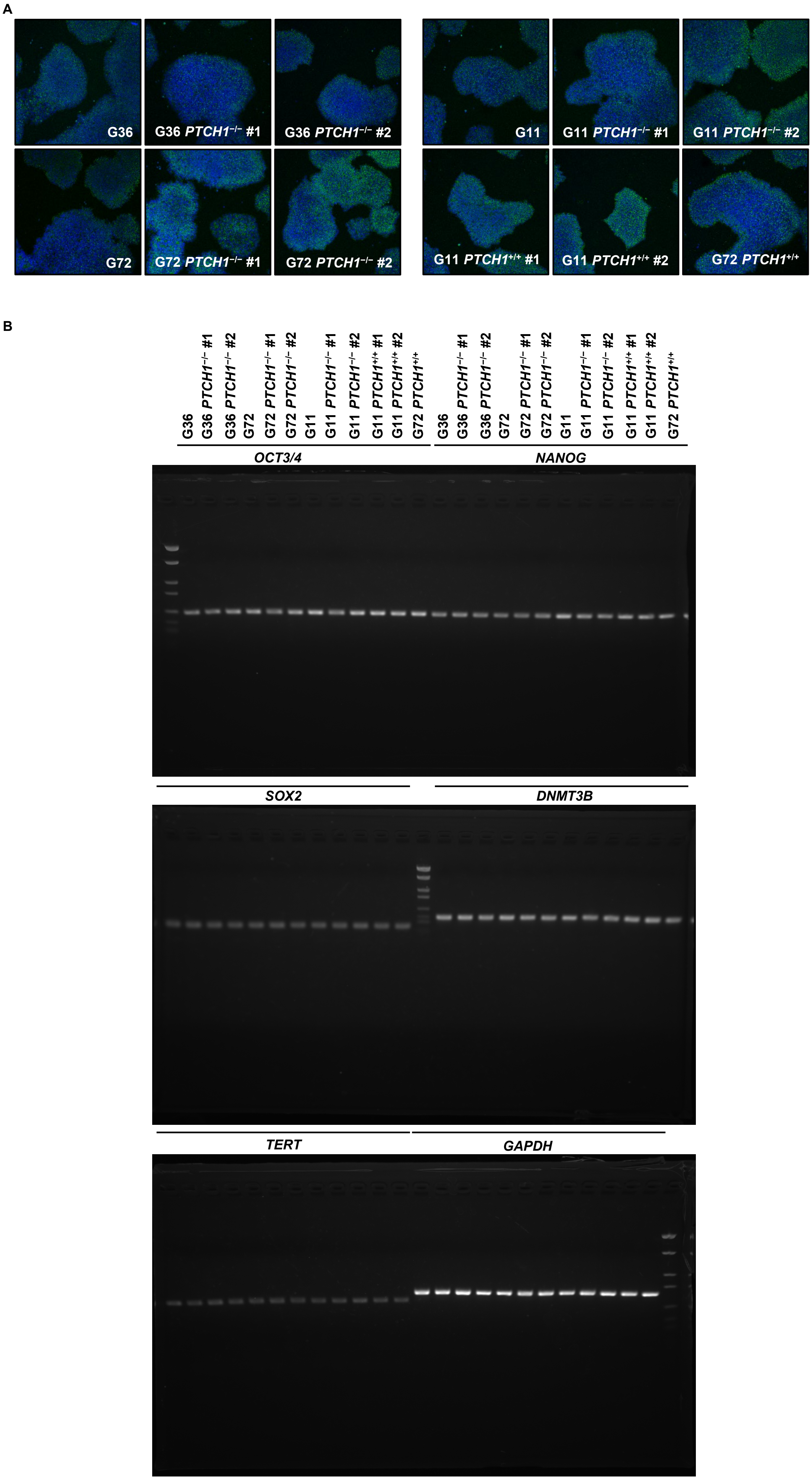

Figure S1. *PTCH1*-edited NBCCS iPSCs expressed pluripotency markers. (A) Immunocytochemical analysis of SSEA4 expression. Blue: DAPI, green: SSEA4. Scale bar: 200 µm. (B) RT-PCR analysis of the expression of the pluripotency markers, *OCT3/4*, *NANOG*, *SOX2*, *DNMT3B*, and *TERT*. *GAPDH* served as the internal control.
