# Supplementary material for: PTCH1-null induced pluripotent stem cells exclusively differentiate into immature ectodermal cells with large areas of medulloblastoma-like tissue
Source: Discov Oncol. 2022 May 27;13:36. doi: 10.1007/s12672-022-00498-x (PMC9135936; doi:10.1007/s12672-022-00498-x)
Supplement: Supplementary file 4 — Supplementary file4 Fig. S2. Whole-slice images and portions of medulloblastomas in PTCH1+/− and PTCH1−/− teratomas. (PDF 1070 KB) [file 12672_2022_498_MOESM4_ESM.pdf]

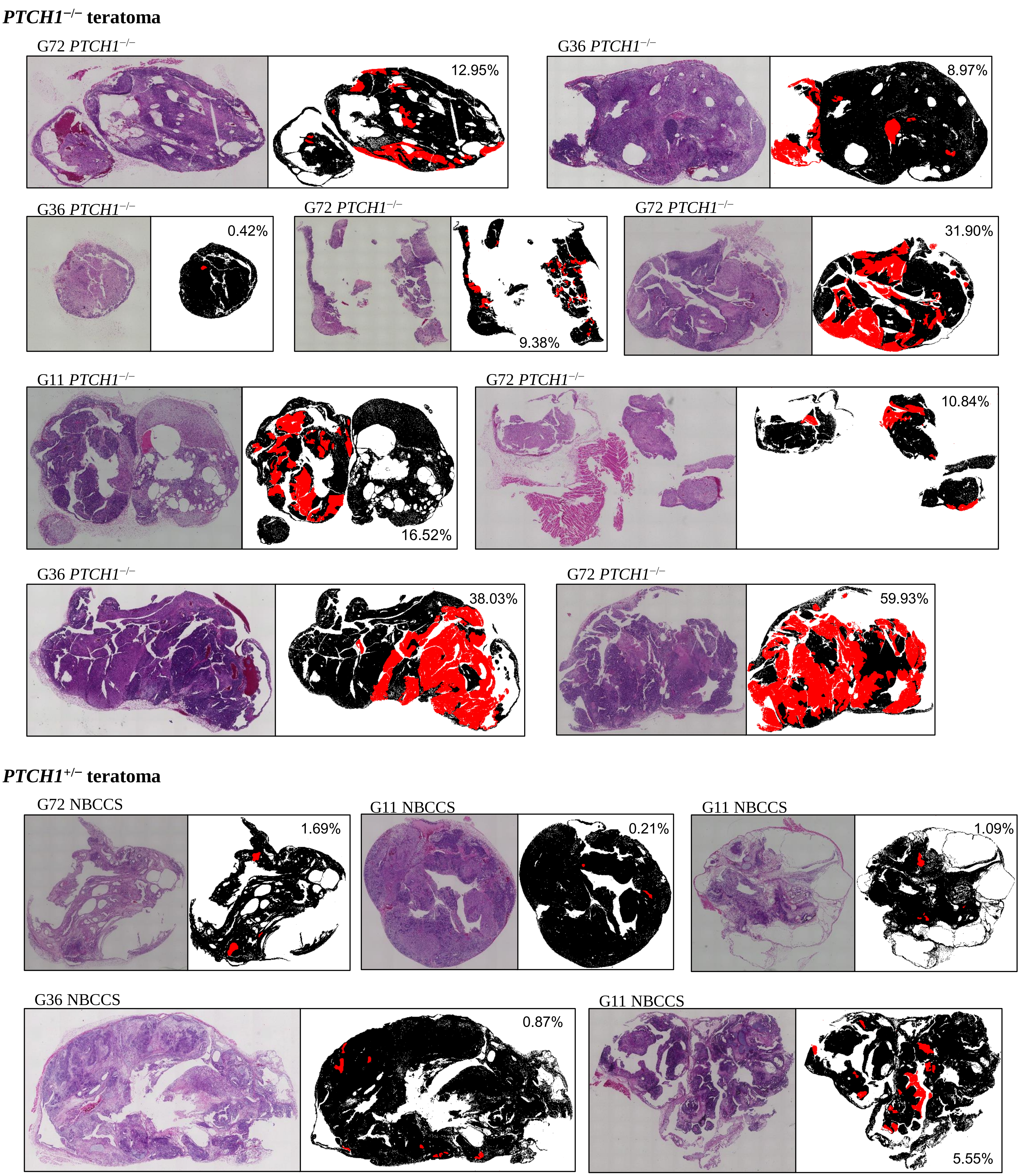

Figure S2. Whole slice images of *PTCH1*$^{-/-}$ and *PTCH1*$^{+/-}$ teratomas.
The left half of each panel represents an image of a HE-stained teratoma slice. In the right half, the area of the teratoma is filled with black from which the apparent tissue from a mouse origin was excluded. Medulloblastoma-like tissue expressing βIII tubulin, synaptophysin, and Ki67 is filled in red. The numbers of pixels in the black and red areas were counted by PixelCounter (https://vector.co.jp/soft/win95/art/se385899.html). The percentage of areas occupied by medulloblastomas is indicated in the right half.
