# Supplementary material for: PTCH1-null induced pluripotent stem cells exclusively differentiate into immature ectodermal cells with large areas of medulloblastoma-like tissue
Source: Discov Oncol. 2022 May 27;13:36. doi: 10.1007/s12672-022-00498-x (PMC9135936; doi:10.1007/s12672-022-00498-x)
Supplement: Supplementary file 5 — Supplementary file5 Fig. S3. The expression of SHH in iPSCs. (PDF 783 KB) [file 12672_2022_498_MOESM5_ESM.pdf]

A

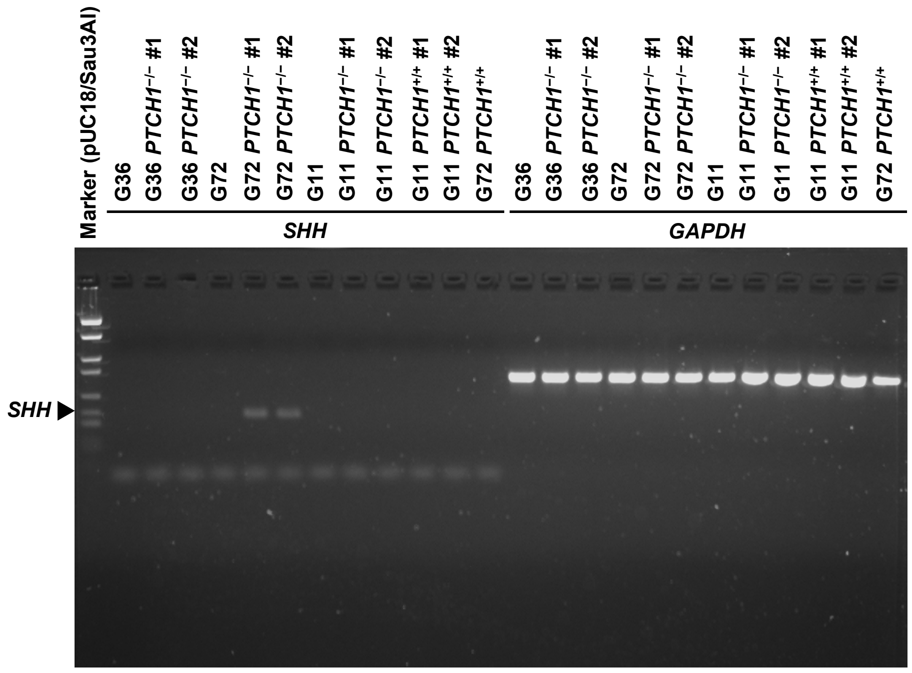

B

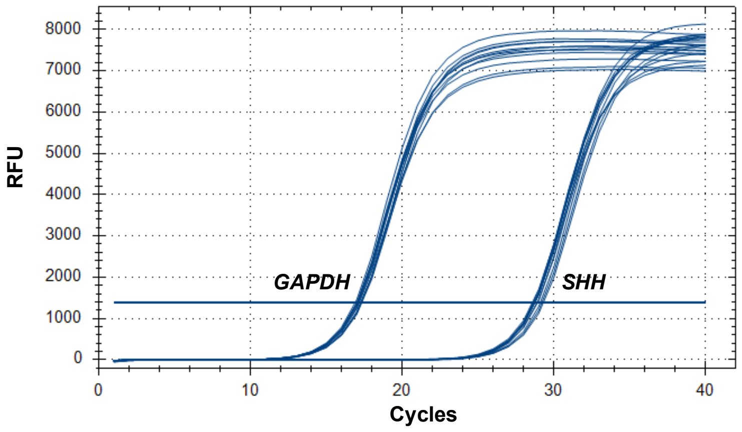

Figure S3. The expression of *SHH* in iPSCs. (A) The expression of *SHH* in iPSC lines. (B) The amplification plot of *SHH* and *GAPDH* in two G72-*PTCH1*$^{-/-}$ lines. The blue horizontal line indicates the threshold line to determine *Ct* value.
